# Supplementary material for: An improved long short term memory network for intrusion detection
Source: PLoS One. 2023 Aug 1;18(8):e0284795. doi: 10.1371/journal.pone.0284795 (PMC10393181; doi:10.1371/journal.pone.0284795)
Supplement: Attachment — Submitted filename: response_to_reviewer 6.docx [file pone.0284795.s001.docx]

**Response to Reviewer 6**

**Article ID:**
PONE-D-22-34491

**Original Article Title:**

An Improved Long Short Term Memory Network For Intrusion Detection

**Re:** Response to reviewers

Dear Editor-in-chief,

Thank you for allowing a resubmission of our manuscript, with an opportunity to address the reviewers’ comments. We appreciate the Editor and the reviewers for taking the time to review our manuscript carefully and for providing valuable feedback to help us improve the quality of the document.

We have made every effort to eliminate all the indicated major and minor inconsistencies and do hope that as a result, the quality of our revised manuscript has improved further. The updated parts are highlighted in red color.

- **There is a relatively good literature review, but still there are some recent relevant articles that should be mentioned, like** [**https://doi.org/10.1007/978-981-19-4831-2_1**](https://doi.org/10.1007/978-981-19-4831-2_1) **or**  [**https://doi.org/10.7717/peerj-cs.956**](https://doi.org/10.7717/peerj-cs.956)**.**
- Authors have included the suggested papers as references [54] and [53], In addition, most related references are also included, such as [47],[48], [59], [34], and [60]. All references mentioned are discussed on page (4), from lines 142 to 147, and on page (5) in lines 168–190.
- **The representation of the candidate solutions should be explained and exemplified.**
- We added a new section “Preliminary Work” on pages (7-10) including all algorithms used in the proposed algorithm. The proposed algorithm was presented in a new section (section 4) to make it clearer, see page (10) and (11).
- **The split of the data set between training, validation and test set should be clearly stated. Also, please clarify what set is used (validation or test?) for calculating the accuracy for the fitness function.**
- In our paper, we used all KDDTrain+ for training, all KDDTest+ and all KDDTest-21 datasets for testing. The training dataset is 80% of NSL KDD dataset and the testing dataset is 20 % of NSL KDD dataset as defined in page (14) starting from line 399.
- We used the testing dataset (i.e., KDDTest+,KDDTest-21) for calculating the accuracy of the fitness function.
- **The authors were very negligent with the text of the manuscript. There are many typos, many words written in capital letter in the middle of the sentences, words that are several times abbreviated, the figures are not well referenced (they appear with ??).**
- The paper has been revised and modified, and all references to figures have been corrected.
